# Trend detection with non-detects in long-term monitoring, a mixed model approach

**DOI:** 10.1007/s10661-023-11285-8

**Published:** 2023-05-12

**Authors:** Martin Sköld

**Affiliations:** 1 Department of Environmental Research and Monitoring, Swedish Museum of Natural History, Stockholm, Sweden; 2 Department of Mathematics, Stockholm University, Stockholm, Sweden

**Keywords:** Contaminant monitoring, Linear mixed model, Censored regression, Trend detection

## Abstract

In long-term monitoring of contaminants in biota, a common approach is to use yearly geometric means of measured concentrations in sampled individuals as a basis for trend analysis. When some or all measurements in a particular year are reported as non-detects, it is unclear how to proceed in calculating the yearly mean. I argue that by casting the problem in terms of a mixed model, non-detects can be accounted for using statistical techniques for censored data. The approach is illustrated using data from the Swedish national monitoring programme for contaminants in biota.

**Supplementary Information:**

The online version contains supplementary material available at 10.1007/s10661-023-11285-8.

## Introduction

Non-detects, experimental values that fall below the level of detection (LOD) of the measurement equipment, are common in environmental monitoring of contaminants. Rather than a fixed contaminant concentration, such values are reported as an upper threshold (i.e., the LOD) by analytic laboratories.

A related threshold is the level of quantification (LOQ), below which values can not be reported within a prescribed accuracy. Reporting practices tend to vary between analytic laboratories, where some report values measured between LOD and LOQ — even if they may be highly uncertain — while others use the LOQ as the lower limit of reporting concentrations.

In the statistical literature, a measurement that is given as an upper bound rather than a fixed value is referred to as an *observation censored from the left*. Even though there is a substantial literature on proper statistical approaches for dealing with censoring — promoted in the environmental literature by in particular Dennis Helsel (e.g., Helsel, 2011, 2010) — simplistic methods like midpoint substitution are still common. A recent review that also lists available statistical software can be found in Shoari and Dubé (2018).

**Fig. 1:**
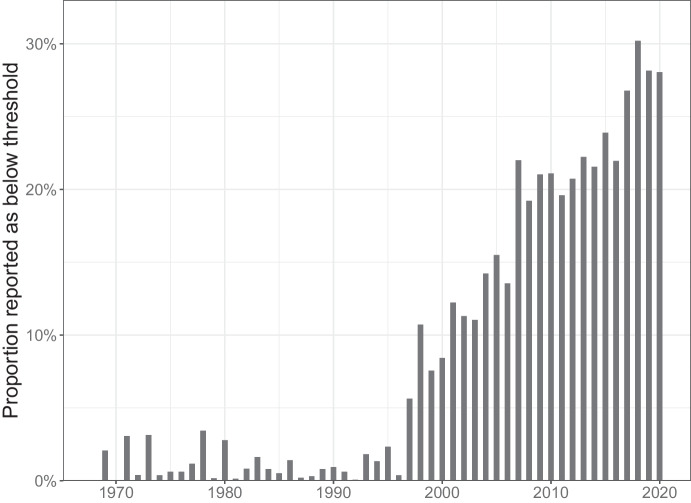
Yearly proportions of measurements reported in the form of an upper bound within the SMCB according to the monitoring programme database (Soerensen & Faxneld, 2020)

While the problem is not new, it has in recent years become more acute in long-term monitoring programmes. Many legacy contaminants show diminishing concentrations in the environment and eventually they reach levels hard to detect. The Swedish national Monitoring programme for Contaminants in marine Biota (SMCB; Soerensen & Faxneld, 2020), coordinated by the Swedish Museum of Natural History, monitors 26 sites along the Swedish coastline for approximately 100 contaminants in a variety of matrices (fish, mussels and bird eggs). This generates several thousands of time series of yearly indices that are examined for trends. Each yearly index is in turn based on a sample of 2–20 individual measurements and for the monitoring year 2020, more than 8000 sample concentrations were measured. Almost a third of these concentrations were censored, i.e., reported as being below a threshold of measurement. This proportion has increased steadily since the mid-1990s (Fig. 1), partly due to decreasing levels of legacy contaminants, and partly due to some laboratories switching from LOD to LOQ as their lower threshold of reporting.

There are at least two aspects that distinguish large-scale long-term monitoring programmes from other contexts involving non-detects. Firstly, there is often a strong focus on *detecting* as opposed to *describing* trends. While describing the magnitude of a trend is by no means unimportant, large-scale programmes are often designed with concepts of power and false positive/type I error rates in mind (Bignert et al., 2004; Fryer & Nicholson, 1993). Secondly, the rules of detection are likely to change over the course of a long-term programme. The SMCB has been running for more than 50 years, during which it has seen changes in measurement equipment, in laboratories performing the analysis, and in reporting standards and guidelines.

It may be argued that focus on trend detection actually works *in favor* of simplistic methods. As long as the censoring mechanism remains consistent over time, an equally time-consistent approach like midpoint substitution will actually not bias the trend estimate *at the position of the null-hypothesis of no trend*. As we will see, the second aspect works in the other direction. When detection thresholds change over time, midpoint substitution is likely to confuse a change in threshold with a change in contaminant load leading to inflated risks of falsely detecting trends.

In this paper I focus on the detection of trends using log-linear regression methods. In particular the case where replication introduces statistical dependence among groups of measurements. I show that the common approach of performing log-linear regression on yearly geometric means is equivalent to fitting a log-linear mixed model to individual observations. This allows us to extend the approach to the censored case, where it is otherwise unclear how the yearly geometric means should be computed. The approach is illustrated using data from the SMCB.

## Methods

Consider a monitoring programme running over $T$ years, $t_1, \ldots , t_T$. In year $t_j$, the concentration of a substance is measured in each of $n_j$ individual samples. Since samples of concentrations are always non-negative, often with a distribution skewed to the right, a common assumption is that this distribution is well described by a log-normal one. In order to cast the problem of detecting a trend in terms of a simple linear regression model with normally distributed residuals, I will hence use log-concentrations as the observational unit. The same results apply on the original scale if concentrations can be assumed normally, rather than log-normally distributed.

In order to introduce the concept of censoring, I start with the case of $n_j=1$, that is where we have measured a single log-concentration each year $t_j$. I denote a measurement by the pair $(y_j, I_j)$, where $I_j$ is an indicator of censoring such that when $I_j=0$, $y_j$ is a measured log-concentration while when $I_j=1$, $y_j$ instead denotes the logarithm of the censoring threshold (e.g., $\log (\text {LOD})$). A model that incorporates a linear yearly trend on the log-scale may now be written as

1 $$\begin{aligned} \begin{aligned} y_j&=\alpha + \beta t_j +\epsilon _j, \text { if }I_j=0,\\y_j&>\alpha + \beta t_j +\epsilon _j, \text { if }I_j=1, \end{aligned} \end{aligned}$$

where I consider the $\epsilon _j$ as independent residuals with an $N(0, \sigma ^2)$-distribution. On the original scale, a yearly relative trend in concentration can now be computed as $100\times (\exp (\beta )-1)\%$. The model described by Eq. (1) is often referred to as the *tobit model* after economist James Tobin, who introduced it in the context of household expenditure data (Tobin, 1958). In the environmental literature it is more commonly called the method of *maximum likelihood*, referring to the way it is usually fitted. I will use the former to distinguish it from other uses of the maximum likelihood principle.

Tobit regression has implementations in major statistical software packages and is often the recommended approach to deal with censored data in the context of linear regression (Thompson & Nelson, 2003; Helsel, 2011). Unfortunately, it can not always be directly extended to the situation with replicate yearly measurements.

### Replicate measurements and between-year variation

When there are replicate measurements in a monitoring year, a statistical issue is whether to base trend analysis on individual measurements or on a yearly index like their mean or median (Fryer & Nicholson, 1993; Gewurtz et al., 2011). The rationale behind regressing on a yearly index rather than individual measurements comes from the assumption of a random between-year variation. Such variation may originate from, e.g., differences in environmental conditions that affect all measurements in a similar manner within a year, due to the fact that it is practically impossible to take a perfectly random sample of the population of interest (e.g., fish in a sea) or due to batch effects in the analyzing laboratory. This will introduce a statistical dependency between individual measurements within a year, a phenomenon often referred to as *pseudoreplication* (Hurlbert, 1984). In this case, the classical assumptions of the linear regression model are violated and the output of statistical software are likely to be misleading unless the dependence is accounted for. Regressing on a single yearly index avoids this problem, but in the presence of censored observations it is not clear how a yearly index should be computed. In particular so when all measurements in a particular year are below the threshold.

Within the SMCB, the problem has historically been handled by replacing censored measurements with *threshold*/$\sqrt{2}$ before an index is computed as the geometric mean (or equivalently the arithmetic mean on the log-scale). Midpoint substitution, where the threshold is divided by 2 rather than $\sqrt{2}$, or removal of censored measurements before computing the index are other common approaches. They all have in common that they will introduce a bias in the yearly index. It is clear that removing values below a threshold will bias the index upwards. For the substitution approaches, the sign of the bias will depend on the threshold as well as the standard deviation of measurements on the log-scale. Given a fixed percentage of censored observations, they will bias the index downwards for small standard deviations and upwards for large. In general there will exist an intermediate range of standard deviations where bias is small, but this will vary between different censoring percentages.

**Fig. 2:**
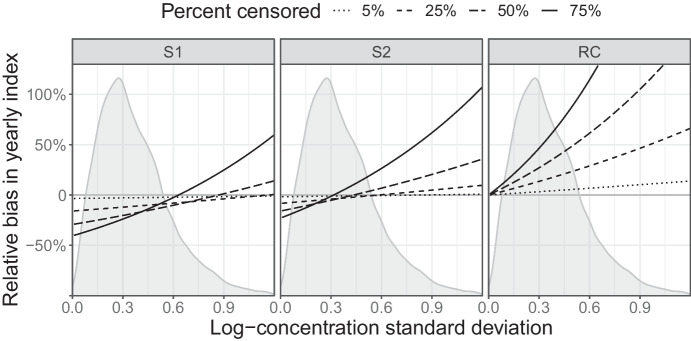
Relative bias (ratio of expected to true value minus one) in yearly index associated with S1: midpoint substitution, S2: $\mathit{threshold}/\sqrt{2}$-substitution and RC: remove censored. The bias has been computed on the log-scale and exponentiated, hence it only approximately reflects bias on the original scale. For the RC approach, it is implicitly assumed that at least one value remains uncensored. Superimposed are density estimates reflecting the relative occurrence of within-year standard deviations in the SMCB (see Supplementary material)

In Fig. 2 I illustrate how the bias in the yearly index depends on the standard deviation of individual log-concentrations typically encountered within the SMCB. Analytical calculations involved in deriving expressions for the bias are presented in the Supplementary material. In particular the bias introduced by removing censored values can be severe even for a moderate level of censoring. We also see that, for the purpose of estimating yearly indices, substituting with $\mathit{threshold}/\sqrt{2}$ works somewhat better than midpoint substitution for the most common standard deviations in the range 0.2 to 0.4.

### A mixed model for uncensored data

In order to approach the problem with likelihood arguments, I propose to explicitly model the yearly variation starting with the case where all concentrations are observed. Denote by $y_{ij}$ the log-concentration measured on the $i$:th individual in the $j$:th monitoring year $t_j$. A model for individual log-concentrations that include random between-year variation may be formulated as

2 $$\begin{aligned} y_{ij} = \alpha + \beta t_j + b_j + \epsilon _{ij}, \end{aligned}$$

where $b_j\sim N(0, s^2)$ represent the between-year variation and $\epsilon _{ij}\sim N(0, \sigma ^2)$ the individual variation, all assumed to act independently of each other. This is a *linear mixed model* (Laird, 1982) in its simplest form, where *mixed* refers to the fact that it contains both fixed (in the form of $\beta t_j$) and random ($b_j$) effects describing between-year variation. When $s=0$, the random effects $b_j$ vanish and we are left with the classical model of simple linear regression.

Linear mixed models are in general fitted using either Maximum-Likelihood (ML) or REstricted Maximum-Likelihood (REML), that for this simple setup only differ in the way they estimate the variance components. The trend estimates will be the same (Harville, 1977). In the case of equal number of yearly replicates, fitting the mixed model using REML is statistically equivalent to simple linear regression on yearly arithmetic means in the sense that both methods give the same estimate of trend and associated standard error (see Supplementary information). In the absence of censoring, the mixed model thus provides a model-based justification for the practice of using yearly means as a base for trend analysis if the number of yearly replicates are roughly equal. Moreover, as a model for individual measurements it provides a unified framework for analyzing data with and without censored measurements.

If the number of yearly replicates are not equal, the yearly arithmetic means will be heteroscedastic and linear regression will, in contrast to the mixed model, not put the optimal weight on each observation. While there is a theoretical advantage of using the mixed model in this situation, differences are expected to be small unless there is a high variation in the number of yearly replicates.

### Extending to the censored case

By introducing indicators of censoring $I_{ij}$, the linear mixed model in Eq. (2) can be extended to cover censored data in complete analogy with the tobit model in Eq. (1). Early development of censored linear mixed models was motivated by medical applications, modelling of HIV viral loads in particular (Pettitt, 1986; Hughes, 1999; Vaida & Liu, 2009), but they have seen recent use also in the environmental literature (e.g., Dodson et al., 2017; Shoari and Dubé, 2017; Munoz et al., 2017) with random effects characterizing differences between, e.g., sites or species. Within the particular framework of trend assessment, mixed models are currently implemented as part of the OSPAR web-tool for contaminants in biota (Rob Fryer, personal communication; Fryer, 2022).

If we denote by $\phi _\sigma$ and $\Phi _\sigma$ the normal density and distribution functions respectively, with standard deviation $\sigma$ and mean zero, the likelihood function of the model can be written as

3 $$\begin{aligned} L(\theta )=&\prod _{j = 1}^T\int \prod _{i = 1}^{n_j}\phi _\sigma (y_{ij}-\alpha -\beta t_{j}-b_j)^{I_{ij}-1} \\&\Phi _\sigma (y_{ij}-\alpha -\beta t_{j}-b_j)^{I_{ij}}\phi _s(b_j)\,db_j, \end{aligned}$$

where $\theta = (\alpha , \beta , \sigma , s)$ denotes the vector unknown parameters. Maximizing this likelihood is challenging since it involves integrals that need numerical evaluation. The EM-algorithm, as implemented in R package lmec (R Core Team, 2020; Vaida & Liu, 2009) is a popular approach for maximizing likelihoods of this type, but I found that computational time of lmec was prohibitively long for high proportions of censoring. Instead, I take advantage of the relatively simple setup with a single random effect per group and approximate the integrals using Gauss-Hermite quadrature (Liu, 1994). My implementation is freely available as an R package mixcens (https://github.com/mskoldSU/mixcens), offering substantial gains in computation time over lmec for many data sets. A small simulation study that compares computational time and numerical results is presented in the Supplementary information.

## Case studies

Out of the full monitoring programme, I have chosen to focus on two scenarios that serve to illustrate issues of bias in trends and inflated risks of falsely detecting trends in simple substitution methods. The methods compared are

- **MM**: The linear mixed model. This is fitted by maximizing Eq. (3) with respect to $\theta$ using a custom-built function in R. Confidence intervals and tests are of Wald-type, based on asymptotic normality and using observed Fisher-information matrices to approximate standard errors.
- **S1**: Midpoint substitution. For this method, concentrations reported as being below a threshold are substituted by $\mathit{threshold}/2$. After substitution, yearly indices are computed as the arithmetic mean of log-concentrations, and the trend is fitted by ordinary linear regression on yearly indices (as implemented by R’s function lm). Confidence intervals and tests are taken unmodified from the software output.
- **S2**: Same as **S1**, though with values below a threshold replaced by $\mathit{threshold}/\sqrt{2}$. This is the approach historically taken by the SMCB.
- **RC**: Remove censored. Values reported as being below a threshold are ignored, remaining values are used to form yearly indices as in **S1**.
- **TM**: Tobit model. This fits the censored regression model Eq. (1) without computing yearly aggregates and hence ignoring potential pseudoreplication (using NADA::cenreg in R; Lee, 2020). Confidence intervals and tests are of Wald-type as with **MM**.

**Fig. 3:**
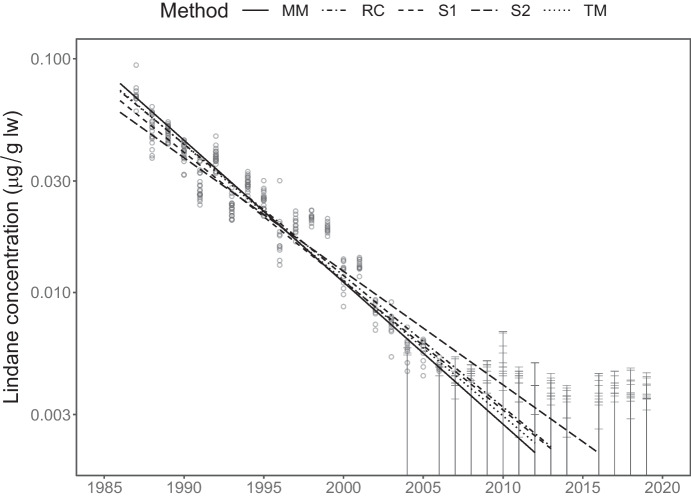
Lindane concentrations measured in herring muscle from Landsort monitoring site together with fitted trends. Circles denote measured concentrations while horizontal bars denote reported levels of quantification (LOQ). Note the logarithmic scale on the *y*-axis

### A legacy contaminant

Lindane ($\gamma$-HCH) is a persistent legacy contaminant that was phased out in Sweden from the 1970s until a complete ban in 1989. Figure 3 shows time series of lindane concentration measured in herring (*Clupea harengus*) muscle at monitoring site Landsort together with fitted trends. From 1987 to 1995, concentrations were measured in 20 sampled individuals which was reduced to 12 individuals in 1996 and onwards. As can be observed in the figure, concentrations decreased in a log-linear fashion until the mid-2000s, where levels reached the limit of quantification. The SMCB contains an additional six time series of lindane measured in this matrix since the late 1980s (more sites have been added later), all showing similar pattern. Other hexachlorocyclohexanes ($\alpha$-HCH and $\beta$-HCH) and dichlorodiphenylethanes (DDT, DDE and DDD) also show similar patterns at many sites. For emerging contaminants, like poly- and perfluoroalkyl substances (PFASs), the situation is reversed; here some contaminants have been retrospectively analyzed since the 1980s resulting in a decade or two of concentrations consistently below LOQ before the first detected concentration.

**Fig. 4:**
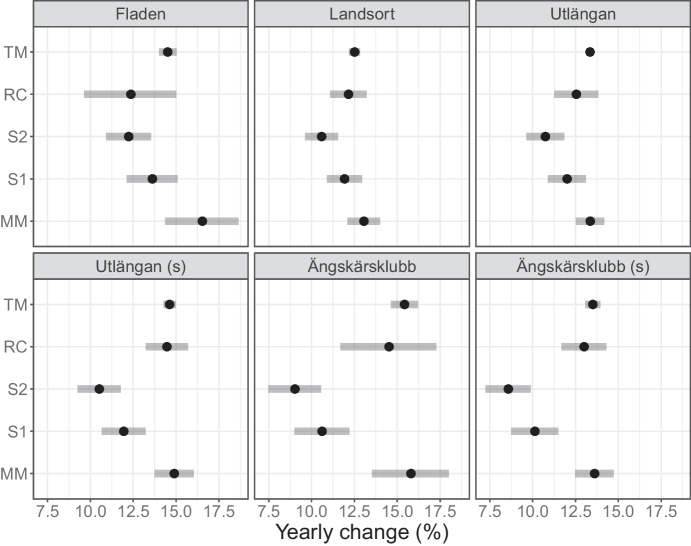
Fitted trends with 95% confidence intervals for seven time series of concentrations of lindane in herring muscle at four sites. Samples are usually collected in autumn, but at sites Utlängan and Ängkärsklubb they are also collected in spring (denoted by (s) in the figure) and treated as separate series

Fitted trend lines in the figure look similar with the exception of method S2, which is more strongly influenced than, e.g., S1 by the censored observations since it substitutes with a value closer to the threshold. In order to get a more detailed view, Fig. 4 illustrates estimated trends with confidence intervals for all seven time series of lindane using each of the five approaches, full tables of results are available in the Supplementary information. The two substitution approaches consistently estimate a somewhat weaker trend than the other methods, since the trend lines are leveraged by censored observations in the end of the series. Estimated variance components from the mixed model in Table 1 suggest that between-year variation accounts for between 40 and 93 percent of the total variance. This confirms that tobit regression, which ignores this source of statistical dependence, attaches too much confidence in its estimates as is reflected by the narrow confidence intervals.

**Table 1:** Variance parameters estimated from linear mixed model for the seven time series of lindane concentrations

Site	*s*	$\sigma$	$s^2/(s^2 + \sigma ^2)$
Ängkärsklubb	0.13	0.16	0.40
Ängkärsklubb (spring)	0.13	0.14	0.45
Fladen	0.38	0.11	0.93
Harufjärden	0.17	0.12	0.67
Landsort	0.19	0.11	0.76
Utlängan	0.17	0.09	0.79
Utlängan (spring)	0.17	0.10	0.73

**Fig. 5:**
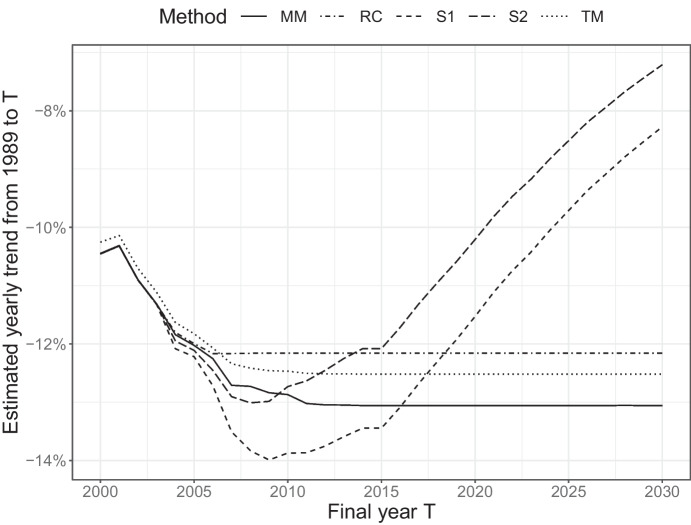
Evolution of trend estimates. Each point corresponds to an estimate of yearly trend based on data from 1983 until T. Data from 2019 and onwards are drawn with replacement from 2009–2018 (all below LOQ)

In order to further illustrate the different approaches, I examine how trend estimates evolve with time, i.e., as more data is included in the model fitting. Figure 5 shows annual estimates of trends at Landsort based on data from 1987 to a final year $T$, where $T$ ranges from 2000 to 2030. Data for yet unobserved years 2020–2030 have been drawn by, for each year, randomly selecting 12 LOQ-values from previous years. Up until 2002 there are no censored observations, for the first three years the substitution methods and the removal method are thus identical. Here, the mixed model and tobit methods differ slightly since they take into account that fewer yearly samples are available from 1996. Since the last value above LOQ was measured in 2008, the removal algorithm produces constant estimates after this year. The mixed model and tobit approaches stabilize a few years later since, under the assumption of a log-linear trend, the fact that the trend-line has decreased below LOQ is already confirmed by earlier observations and new observations of an upper limit provide negligible information. The two substitution approaches, on the other hand, estimates weaker and weaker trends, approaching a zero asymptote.

Of course, there is no way to formally test the assumption of a log-linear trend beyond the last observed measurement. When using predicted concentrations beyond this point, the usual caution with extrapolation applies.

### From LOD to LOQ

**Fig. 6:**
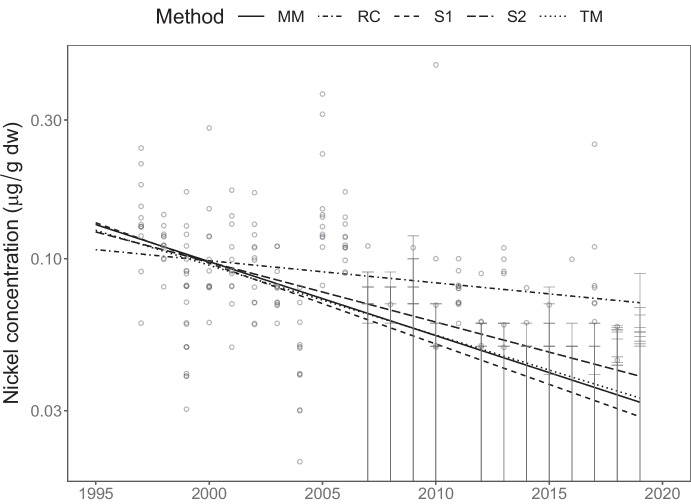
Nickel concentrations measured in herring liver from Fladen monitoring site together with fitted trends. Circles denote measured concentrations while horizontal bars denote reported levels of quantification. Note the logarithmic scale on the *y*-axis

Principles of how to report concentrations that are hard to quantify may vary considerably between laboratories and be highly dependent on analytic methods. For the analysis of metals, the SMCB underwent a change of analytic laboratory in 2007. The change is clearly visible in Fig. 6, showing a time series of nickel concentrations in herring liver, due to different reporting practices of the two laboratories; the first laboratory reported measurements even if they were between LOD and LOQ, while the laboratory employed from 2007 onward used the LOQ as a lower reporting threshold. The fact that a few values above LOQ are still reported in recent years suggests that the initial decrease has halted, possibly because concentration has reached a naturally occurring level.

I use this example as inspiration for a simulation study involving no real trend but a change of reporting practice. The purpose is to evaluate accuracy of trend detection for the five approaches, in particular whether they attain the correct *level of significance*, corresponding to the rate of Type I errors associated with falsely detecting a trend from random noise. Statistical tests are in general calibrated towards a fixed significance level, e.g., 5%, meaning that if repeatedly and independently applied to random data without a trend, they will falsely detect a trend in on the average 1 case out of 20. Often it is difficult to calibrate a test to *exactly* attain the prescribed significance level. In a large-scale monitoring context, where false alarms may be costly, it is crucial that the true significance level does not greatly exceed the prescribed.

In my simulations I consider detecting a 20-year trend based on 10 individuals sampled each year and where a threshold (LOQ) is introduced after the tenth year. A situation similar to the analysis of metals within the SMCB. Parameters that will impact the results are the proportion of values censored, the between-year standard deviation $s$ and the individual standard deviation $\sigma$. I choose to fix proportion censored in $\{25\%, 50\%, 75\%\}$ and between-year standard deviation $s \in \{0, 0.1, 0.3\}$, while residual standard deviation $\sigma$ is allowed to vary freely between 0.1 and 0.7. This corresponds to values typically seen within the SMCB. My aim is not to provide a full understanding of the performance of methods under a variety of conditions, but rather to illustrate the erratic behavior of the simpler methods.

**Fig. 7:**
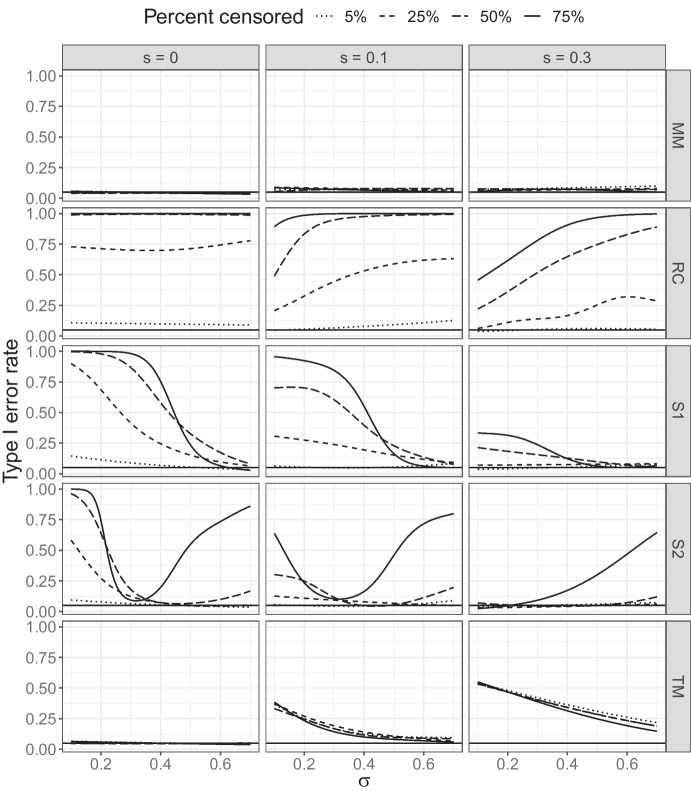
Approximate risks of falsely detecting a trend from random noise under a range of scenarios. Deviations from the prescribed risk of 0.05 are due to bias in trend estimates and/or deflated estimates of parameter uncertainty

Figure 7 shows how the actual type I error rate compares to the prescribed 0.05. Note that results are based on simulations and subject to a small Monte-Carlo error, full details of the simulations are given in the online code repository.

The mixed model (MM) shows the most stable behavior among the methods, with type I errors ranging from 0.04 to 0.08 close to the prescribed 0.05. The difference is small enough to be influenced by Monte-Carlo error and more simulations are needed to confirm any patterns seen in the figure. It does seem to reject somewhat more often than prescribed, which is to be expected due to the simple Wald-type test applied. Even better results may be obtained using a likelihood-ratio approach at some additional computational expense.

The two substitution approaches (S1 and S2) on the other hand detect false trends in up to 100% of the simulated data sets under some scenarios, and are highly sensitive to the value of $\sigma$. As in Fig. 2, S2 performs the best around $\sigma =0.3$ which is a commonly seen value in the SMCB. This further confirms its superiority to midpoint substitution (S1) within this monitoring programme.

The Tobit model (TM) gives near unbiased estimates of trend (Fig. S2) and leads to accurate type I errors in the absence of between-year variation ($s=0$). For $s=0.1$ and $s=0.3$ it again suffers from underestimating parameter uncertainty, leading to inflated type I errors.

Finally, removing censored observations (RC) works poorly for all scenarios in this setup.

## Discussion

Linear and generalized linear mixed models have become increasingly popular in biology during the past decade (Zuur et al., 2009; Bolker et al., 2009), allowing flexible strategies to deal with statistical dependence in data. I show how they can be used in a long-term environmental monitoring context, to deal with a common issue of pseudoreplication in combination with values reported as an upper limit.

The gains come as reduced bias in trend estimates and greatly reduced risks of falsely inferring trends when no trend exists. The price to be paid comes in the form of increased computational complexity. While fitting a simple linear regression is essentially instant, the mixed models involving censored observations presented in the paper took up to a few seconds to fit. This may not matter much for fitting a few trends, but within a large-scale monitoring programme with thousands of time series it may be inconvenient. Here it is reassuring that if there are no censored observations and equal number of yearly observations, the same model can be fitted using simple log-linear regression on yearly geometric means. This is the recommended approach unless there is particular interest in disentangling *s* and $\sigma$.

Using the mixcens package reduced computation time considerably in comparison to lmec. For example, fitting the trend in Fig. 4 took less than 2 s with mixcens and 190 s with lmec on a MacBook Pro using default settings. However, in comparison to mixcens, lmec can fit a much wider range of mixed model structures.

## Conclusions

In long-term monitoring programmes, the presence of non-detects requires extra care when assessing trends in time series data. Simplistic substitution approaches, e.g., using half the detection limit as a surrogate measurement value, are likely to cause bias in trend estimates and highly inflated false discovery rates. Using a statistical approach based on linear mixed models, the problems are resolved at the price of additional computational complexity.

## Supplementary Information

Below is the link to the electronic supplementary material.

Supplementary file1 (PDF 236 kb)

## Data Availability

Code and data used for this article are available online at https://github.com/mskoldSU/mixcens_paper.

## Declarations

The author has read, understood, and complied as applicable with the statement on “Ethical responsibilities of Authors” as found in the Instructions for Authors and are aware that with minor exceptions, no changes can be made to authorship once the paper is submitted.
