# Supplementary material for: Trend detection with non-detects in long-term monitoring, a mixed model approach
Source: Environ Monit Assess. 2023 May 12;195(6):663. doi: 10.1007/s10661-023-11285-8 (PMC10182149; doi:10.1007/s10661-023-11285-8)
Supplement: Supplementary file 1 — Supplementary file1 (PDF 236 kb) [file 10661_2023_11285_MOESM1_ESM.pdf]

Supplementary information: Trend detection with non-detects in long-term monitoring, a mixed model approach

Martin Sköld[1,2*]

[1*]Department of Environmental Research and Monitoring, Swedish Museum of Natural History, Sweden.
[2]Department of Mathematics, Stockholm University, Sweden.

Corresponding author e-mail: martin.skold@nrm.se;

1 Bias in yearly indices

In order to derive the bias in yearly indices depicted in Figure 2, suppose the true concentration is equal to Y, where $X = \log(Y) \sim N(\mu, \sigma^2)$. If we replace concentrations less than L by L/d, then observed concentration equals $\tilde{Y} = \exp(\tilde{X}) = Y\mathbf{1}(Y \geq L) + (L/d)\mathbf{1}(Y < L)$. The bias on the log-scale now equals

$$\begin{aligned} b(\sigma) &= E(\tilde{X}) - \mu = E(X\mathbf{1}(X \geq \log(L)) + \log(L/d)\mathbf{1}(X < \log(L))) \\ &= \phi(\log(L)/\sigma)\sigma + (\log(L) - \log(d))\Phi(\log(L)/\sigma), \end{aligned}$$

where ϕ and Φ denotes the standard normal density and distribution functions respectively. In Figure 2, the graphs correspond to $\exp(b(\sigma)) = \exp(E(\tilde{X}))/\exp(\mu)$. Note that this is the exponentiated bias on the log-scale rather than the ratio $E(\tilde{Y})/E(Y)$.

1.1 Standard deviations in Figure 2

Standard deviations used for the density estimate in Figure 2 are computed for each combination of year, site, species and contaminant with at least four uncensored and distinct values. If no concentrations are

Table 1 Estimates of intercept and trend (for log-concentrations) for all Lindane sites

Location	term	estimate	std.error	p-value
Ängskärsklubb	(Intercept)	339	27.0	< 0.01
	year	-0.17	0.013	< 0.01
Ängskärsklubb (spring)	(Intercept)	288	13.3	< 0.01
	year	-0.15	0.007	< 0.01
Fladen	(Intercept)	356	26.0	< 0.01
	year	-0.18	0.013	< 0.01
Harufjärden	(Intercept)	282	18.8	< 0.01
	year	-0.14	0.009	< 0.01
Landsort	(Intercept)	275	11.2	< 0.01
	year	-0.14	0.006	< 0.01
Utlängan	(Intercept)	282	9.7	< 0.01
	year	-0.14	0.005	< 0.01
Utlängan (spring)	(Intercept)	318	13.8	< 0.01
	year	-0.16	0.007	< 0.01

censored, they are estimated as the empirical standard deviations of log-concentrations. When censored concentrations are present, standard deviations are estimated by maximum-likelihood under a censored normal model using `EnvStats::enormCensored` in R (Millard (2013)).

2 All Lindane sites

In Table 1, estimates of trend and intercept is provided for all Lindane sites, corresponding to Figure 4 in the article. Code for generating the table is available at the code repository https://github.com/mskoldSU/mixcens_paper.

3 Equivalence of mixed model and yearly mean aggregation

With $Var(b_i) = s^2$ and $Var(\epsilon_{ij}) = \sigma^2$, the marginal distribution of vector y_i is $N(\mu_i, \Sigma)$, where $\mu_i = X_i(\alpha, \beta)^T$ and X_i is the $d \times 2$ matrix with all rows equal $(1, t_i)$ and $\Sigma = \sigma^2 I + s^2 J$. Here I is the $d \times d$ identity matrix and J the matrix of ones of the same dimension. We may now write the likelihood as

$$
\begin{aligned}
L(\theta) &\propto \prod_{i=1}^{n} \Sigma^{-1/2} \exp(-(y_i - \mu_i)^T \Sigma^{-1} (y_i - \mu_i)/2) \\
&= \prod_{i=1}^{n} \Sigma^{-1/2} \exp(-\frac{1}{2\sigma^2}((y_i - \mu_i)^T I (y_i - \mu_i) - \frac{s^2}{\sigma^2 + ds^2}(y_i - \mu_i)^T J (y_i - \mu_i)))
\end{aligned}
$$

$$
\begin{aligned}
&= \prod_{i=1}^{n} \Sigma^{-1/2} \exp\Big(-\frac{1}{2\sigma^2}\Big(\sum_{j=1}^{d}(y_{ij}-\mu_i)^2 - \frac{d^2 s^2(\bar{y_i}-\mu_i)^2}{\sigma^2+ds^2}\Big)\Big) \\
&= \prod_{i=1}^{n} \Sigma^{-1/2} \exp\Big(-\frac{1}{2\sigma^2}\Big(\sum_{j=1}^{d}(y_{ij}^2-\bar{y_i}^2) + d(\bar{y_i}-\mu_i)^2 - \frac{d^2 s^2(\bar{y_i}-\mu_i)^2}{\sigma^2+ds^2}\Big)\Big) \\
&= \frac{1}{d^{n/2}(\sigma^2)^{n(d-1)/2}} \exp\Big(-\frac{1}{2\sigma^2}\sum_{i=1}^{n}\sum_{j=1}^{d}(y_{ij}^2-\bar{y_i}^2)\Big) \\
&\quad\times \frac{1}{(\sigma^2/d+s^2)^{n/2}} \exp\Big(-\frac{1}{2(\sigma^2/d+s^2)}\sum_{i=1}^{n}(\bar{y_i}-\mu_i)^2\Big) \\
&= g(y,\sigma) f(\bar{y},\tilde{s},\mu)
\end{aligned}
$$

where we in the second last step used that $\det(\sigma^2 I + s^2 J) = (1 + s^2 d/\sigma^2)(\sigma^2)^d$ and where $\tilde{s} = \sqrt{\sigma^2/d + s^2}$. This shows that the likelihood, viewed as a function of (α, β), is proportional to f which only depends on data through $\bar{y} = (\bar{y_1}, \ldots, \bar{y_n})$. Hence, there is no loss of information about (α, β) when aggregating data to yearly means. Further, f is proportional to the likelihood obtained when applying simple linear regression to yearly means, hence estimates of (α, β) are identical.

4 Comparison of `mixcens` and `lmec`

Since `mixcens` and `lmec` employ different numerical methods for maximizing the likelihood, their results will differ slightly depending on the settings of the underlying algorithms. This includes e.g. starting values, stopping rules for assessing convergence and the location/number of quadrature points. In order to ensure that results are comparable, I performed a basic simulation study involving 120 simulated data sets. Each data set consisted of

- 10 years of data with 10 measurements each year
- a yearly increasing trend of 5%
- a censoring percentage selected from $\{5\%, 25\%, 50\%, 75\%\}$
- a residual standard deviation σ selected in the range $[0.1, 0.7]$
- a random effect standard deviation s selected from $\{0, 0.1, 0.3\}$

Trend estimates and algorithm timings are presented in Figures 1 and 2. While there are small numerical differences, mean absolute difference over simulations is just 0.0003 which suggests the methods are for practical purposes identical. Timing shows that `mixcens` remains relatively stable across scenarios while computation time of `lmec` increase drastically as the proportion of censored observations increase. Note that relative performance may be highly sensitive to algorithm settings.

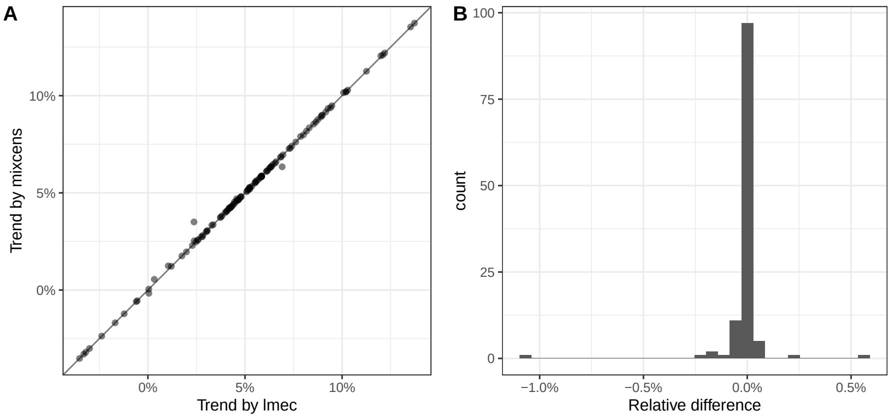

Fig. 1 A: Yearly trend estimates based on `lmec` and `mixcens` with default settings. B: Histogram of relative differences in trend estimates based on `lmec` and `mixcens` computed as $\exp(\beta_{\texttt{lmec}} - \beta_{\texttt{mixcens}}) - 1$ for the 120 simulated data sets

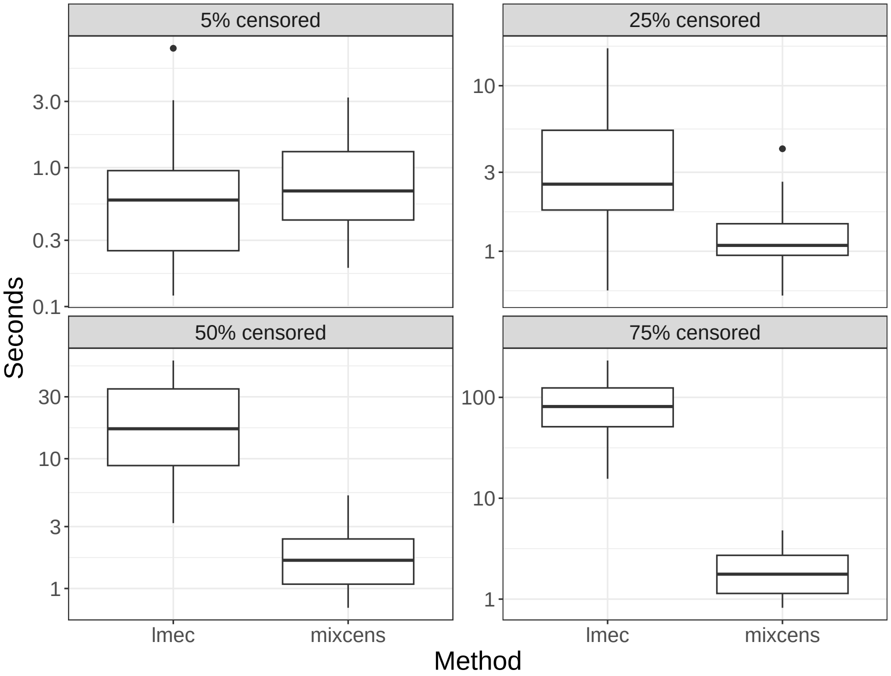

Fig. 2 Timings in seconds of `lmec` and `mixcens` based on 120 simulated data sets

References

Millard, S.P. (2013). *Envstats: An r package for environmental statistics.* New York: Springer. Retrieved from https://CRAN.R-project.org/package=EnvStats
